# High-resolution genome-wide association study pinpoints metal transporter and chelator genes involved in the genetic control of element levels in maize grain

**DOI:** 10.1093/g3journal/jkab059

**Published:** 2021-03-02

**Authors:** Di Wu, Ryokei Tanaka, Xiaowei Li, Guillaume P Ramstein, Suong Cu, John P Hamilton, C Robin Buell, James Stangoulis, Torbert Rocheford, Michael A Gore

**Affiliations:** 1 Plant Breeding and Genetics Section, School of Integrative Plant Science, Cornell University, Ithaca, NY 14853, USA; 2 Institute for Genomic Diversity, Cornell University, Ithaca, NY 14853, USA; 3 College of Science and Engineering, Flinders University, Bedford Park, SA 5042, Australia; 4 Department of Plant Biology, Michigan State University, East Lansing, MI 48824, USA; 5 Department of Agronomy, Purdue University, West Lafayette, IN 47907, USA

**Keywords:** genome-wide association study, whole-genome prediction, elements, grain, maize

## Abstract

Despite its importance to plant function and human health, the genetics underpinning element levels in maize grain remain largely unknown. Through a genome-wide association study in the maize Ames panel of nearly 2,000 inbred lines that was imputed with ∼7.7 million SNP markers, we investigated the genetic basis of natural variation for the concentration of 11 elements in grain. Novel associations were detected for the metal transporter genes *rte2* (*rotten ear2*) and *irt1* (*iron-regulated transporter1*) with boron and nickel, respectively. We also further resolved loci that were previously found to be associated with one or more of five elements (copper, iron, manganese, molybdenum, and/or zinc), with two metal chelator and five metal transporter candidate causal genes identified. The *nas5* (*nicotianamine synthase5*) gene involved in the synthesis of nicotianamine, a metal chelator, was found associated with both zinc and iron and suggests a common genetic basis controlling the accumulation of these two metals in the grain. Furthermore, moderate predictive abilities were obtained for the 11 elemental grain phenotypes with two whole-genome prediction models: Bayesian Ridge Regression (0.33–0.51) and BayesB (0.33–0.53). Of the two models, BayesB, with its greater emphasis on large-effect loci, showed ∼4–10% higher predictive abilities for nickel, molybdenum, and copper. Altogether, our findings contribute to an improved genotype-phenotype map for grain element accumulation in maize.

## Introduction

Elements are important in every aspect of organismal development. In higher plants, at least 20 elements are involved in key biological functions (Mengel and Kirkby 2001). To maintain elemental homeostasis, plants require the activities of metal transporters, chelators, and signaling pathways for the regulation of optimal uptake, transport, and storage of metal ions (Clemens 2001). The complex network responsible for elemental accumulation in various plant organs and tissues such as physiologically mature seed is coordinated at the genetic level, but it can be perturbed by alterations in the soil chemical environment, plant architecture, physiology, and metabolism (Baxter 2009). However, the genetic underpinnings of the biological processes that regulate elemental uptake, transport, and storage have yet to be fully elucidated in model plants and crop species.

Maize (*Zea mays* L.) is a globally important staple crop, serving as a critical source of calories in Sub-Saharan Africa and Latin America (FAOSTAT 2018). However, the declining soil fertility of farming systems contributes in part to the unrealized potential yield of maize in these geographies (Dixon *et al.* 2001). Not only does the deficiency or excess of one or more key elements in the soil limit maize plant productivity (ten Berge *et al.* 2019), but it also has implications for human nutrition if this causes an unfavorable elemental profile in the maize grain (Graham and Welch 1996; Welch 2002; Welch and Graham 2004). This could pose serious malnutrition-related health problems in a maize-based diet because such a diet may not provide the recommended dietary allowances of micronutrients such as iron (Fe) and zinc (Zn) (Welch and Graham 2002). The development of crop varieties with improved nutritional quality through plant breeding, a strategy known as “biofortification,” has the potential to sustainably address micronutrient deficiencies in developing nations (Diepenbrock and Gore 2015; Bouis and Saltzman 2017).

Depending on the element and plant species, elements accumulated in seed could originate from direct root uptake or remobilization from senescing tissues through the involvement of transporters and chelators (Waters and Sankaran 2011). Several metal transporter and chelator protein families, such as METAL TOLERANCE PROTEIN (MTP), NATURAL RESISTANCE-ASSOCIATED MACROPHAGE PROTEIN (NRAMP), NICOTIANAMINE SYNTHASE (NAS), YELLOW STRIPE-LIKE (YSL), and ZINC-REGULATED TRANSPORTER (ZRT)/IRON-REGULATED TRANSPORTER (IRT)-LIKE PROTEIN (ZIP), have been bioinformatically identified in the genomes of Arabidopsis [*Arabidopsis thaliana* (L.) Heynh.], rice (*Oryza sativa* L.), maize and other plant species, yet only a subset from each family have been functionally characterized (Whitt *et al.* 2020). Many metal transporters and chelators have broad substrate specificity (Axelsen and Palmgren 2001), making it difficult to infer their primary roles with homology-based approaches. In maize, only a few metal transporter genes have been functionally studied including *rotten ear1* (*rte1*), *rte2*, and *tassel-less1* (*tls1*) for boron (B) (Chatterjee *et al.* 2014, 2017; Durbak *et al.* 2014), *yellow stripe1* (*ys1*) and *ys3* for Fe (Von Wiren *et al.* 1994; Chan-Rodriguez and Walker 2018), and *ysl2* (Zang *et al.* 2020) and *zip5* (Li *et al.* 2019) for Fe and Zn. Of these, transgenic maize overexpressing *zip5* with an endosperm-specific promoter was shown to accumulate higher levels of Fe and Zn in grain (Li *et al.* 2019).

Genetic mapping approaches offer another opportunity to identify the largely unknown genes responsible for elemental concentration in maize grain. There have been a number of linkage analysis studies that have used biparental populations to identify quantitative trait loci (QTL) associated with an elemental concentration in maize grain, especially for Fe and Zn (Lung’aho *et al.* 2011; Šimić *et al.* 2012; Qin *et al.* 2012; Baxter *et al.* 2013; Jin *et al.* 2013; Gu *et al.* 2015; Asaro *et al.* 2016; H. Zhang *et al.* 2017; Ziegler *et al.* 2017; Fikas *et al.* 2019). However, the biparental populations used in these studies did not provide gene-level mapping resolution due to the limited number of recent recombination events (Zhu *et al.* 2008; Myles *et al.* 2009). Thus, the causal genes presumably residing in the large QTL intervals with low resolution could not be conclusively identified.

Genome-wide association studies (GWAS) that exploit the extensive phenotypic variation and ancient recombination of many individuals comprising a diversity population (association panel) offer higher mapping resolution to dissect complex traits than biparental mapping populations (Myles *et al.* 2009; Lipka *et al.* 2015). A total of 46 marker-trait associations for the concentration of Zn and Fe in grain were identified in a tropical maize association panel (Hindu *et al.* 2018). Some of these associations were independently supported by separate QTL analyses in biparental populations. However, the association signals were not definitively resolved to causal genes. Through joint-linkage (JL) analysis and GWAS in the US maize nested association (NAM) panel, Ziegler *et al.* (2017) identified six high confidence candidate genes underlying association signals for four elements (manganese, Mn; molybdenum, Mo; phosphorus, P; and rubidium, Rb), but not all signals for these and other elements could be unambiguously mapped to single genes. Overall, the promise of GWAS for identifying the causal genes responsible for elemental accumulation in maize grain has yet to be fully realized, but efforts could be improved with the use of larger, more diverse association panels which have been densely genotyped.

When GWAS is employed to elucidate the molecular genetic basis of phenotypes, the significantly associated markers tend to be those in strong linkage-disequilibrium (LD) with causal loci of large effect (Myles *et al.* 2009). Therefore, if a phenotype is genetically controlled by mostly small-effect loci, the heritable fraction of a phenotype may not be completely explained by GWAS-detected loci alone. If this occurs, genomic prediction models that employ all available genome-wide markers to account for a range of small to large marker effects across the entire genome (*i.e.*, whole-genome prediction, WGP) could be used to improve trait prediction accuracy (Meuwissen *et al.* 2001; Gianola *et al.* 2009; de Los Campos *et al.* 2013). Furthermore, trained WGP models are used in genomic selection to increase genetic gain per unit of time when breeding for phenotypes having polygenic inheritance, as marker-assisted selection is better suited for Mendelian and oligogenic traits (Lorenz *et al.* 2011; Desta and Ortiz 2014; Owens *et al.* 2014). To our knowledge, WGP models have only been evaluated on elemental grain phenotypes of wheat (*Triticum aestivum* L.) (Velu *et al.* 2016; Manickavelu *et al.* 2017; Alomari *et al.* 2018) and only Zn for maize (Guo *et al.* 2020; Mageto *et al.* 2020).

In our study, a maize inbred association panel consisting of 1,813 individuals imputed with ∼7.7 million SNP markers was used for the genetic dissection and prediction of natural variation for elemental concentration in grain. The objectives of our study were to (i) assess the extent of phenotypic variation and heritability of 11 elemental grain phenotypes, (ii) conduct a GWAS to identify candidate causal genes controlling variation for 11 elemental phenotypes in maize grain, (iii) compare detected GWAS signals with genetic mapping results from the U.S. maize NAM panel, and (iv) evaluate the predictive abilities of two WGP models having different assumptions of the underlying genetic architecture for the elemental grain phenotypes.

## Materials and methods

### Plant materials and experimental design

We evaluated more than 2,400 maize inbred lines from the North Central Regional Plant Introduction Station association panel (hereafter, Ames panel) (Romay *et al.* 2013) at Purdue University’s Agronomy Center for Research and Education in West Lafayette, IN, on Raub silt loam (fine-silty, mixed, superactive, and mesic Aquic Argiudolls) and Chalmers silty clay loam (fine-silty, mixed, superactive, and mesic Typic Endoaquolls) soils in 2 consecutive years (2012–2013). A single replicate of the entire experiment was grown in each of the 2 years following a design that has been previously described in Owens *et al.* (2019). Briefly, the maize inbred lines were partitioned into six sets according to their flowering time, with each set arranged as a 20 × 24 incomplete block design. Within a set, each incomplete block was augmented with the random positioning of a B73 plot (experiment-wide check) and two plots of a set-specific check. Experimental units were one-row plots that had a length of 3.81 m, with ∼15 plants per plot. The physiologically mature grain from the hand-harvested, dried, and shelled self-pollinated ears (at most six) of each harvestable plot were bulked to generate a representative, composite sample for element analysis.

### Phenotypic data analysis

We ground 4,406 grain samples weighing 10 g each from 2,177 inbred lines and a separate set of 11 repeated check lines with a Retsch ZM200 mill (Retsch, Haan, Germany). For inductively coupled plasma mass spectrometry (ICP-MS) analysis, ∼0.3 g of each ground sample, which had been oven dried at 80°C for 4 h to remove remaining moisture, was acid-digested in a closed tube as described in Wheal *et al.* (2011). Elemental concentrations of samples were measured using ICP-MS (7500x; Agilent, Santa Clara, CA) according to the method of Palmer *et al.* (2014). The 18 quantified elements were aluminum (Al; for only monitoring contamination with soil), arsenic (As), boron (B), calcium (Ca), cadmium (Cd), cobalt (Co), copper (Cu), Fe, potassium (K), magnesium (Mg), Mn, Mo, sodium (Na), nickel (Ni), P, lead (Pb), selenium (Se), and Zn in μg g^−1^ on a dry weight basis. In each of 10 digestion batches, a blank and a certified reference material (CRM; NIST 8433 corn bran) were added for quality assurance. In addition, 6 to 7 experimental samples were replicated twice within each batch, allowing the assessment of technical (measurement) error. Technical replicate sample pairs with a relative standard deviation >10% were removed, which resulted in the removal of three inbred lines. Samples (0.8%) with Al present at >5 μg g^−1^ were considered to have unacceptable levels of purported soil contamination (Yasmin *et al.* 2014), thus resulting in the removal of an additional eight inbred lines from the dataset.

To improve the quality of the resultant dataset of 4,351 samples from the remaining 2,166 inbred lines and separate set of 11 repeated check lines, we assessed phenotypes for missing values due to the limit of detection (LOD) for ICP-MS. The levels of Ca and Ni were below the LOD for 1.98% and 18.30% of samples, respectively. Separately for each of these two elements, a μg g^−1^ value was approximated for the missing value of each of these samples by imputing a uniform random variable ranging from 0 to the minimum ICP-MS detection value for the given element within each year (Lubin *et al.* 2004; Lipka *et al.* 2013). Given the potential for biased results (Lubin *et al.* 2004), we excluded six elements (As, Cd, Co, Na, Pb, and Se) that had more than 70% of samples with a concentration below the LOD for ICP-MS.

**Figure 1:**
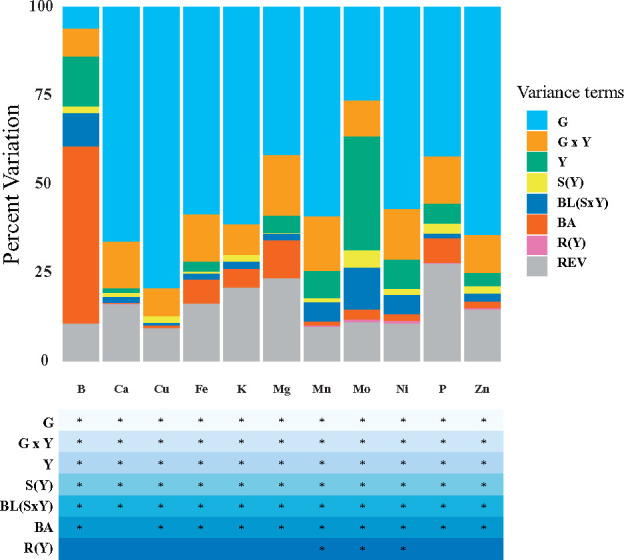
Sources of variation for 11 elemental grain phenotypes in the Ames panel. The phenotypic variance was statistically partitioned into the following components: genotype (G), genotype-by-year interaction (G × Y), year (Y), set within year [S(Y)], block within set within year [BL(S × Y)], inductively coupled plasma mass spectrometry (ICP-MS) batch (BA), row within year [R(Y)], and residual error variance (REV). Variance component estimates were calculated for all random effects from the full model Equation 1 of Owens *et al.* (2019). The table below indicates which random effects were significant (*) according to a likelihood ratio test (α = 0.05).

We screened the generated dataset of 11 elemental phenotypes without missing values from the 2,166 inbred lines and separate set of 11 repeated check lines for significant outliers according to the procedure of Owens *et al.* (2019). Briefly, the full mixed linear model (Equation 1) of Owens *et al.* (2019) was fitted for each elemental phenotype in ASReml-R version 3.0 (Gilmour *et al.* 2009). The model terms included check as a fixed effect and genotype (noncheck line), year, genotype-by-year (G × Y) interaction, set within year, plot grid row within year, incomplete block within set within year, and ICP-MS batch as random effects. Studentized deleted residuals (Neter *et al.* 1996) produced from these mixed linear models were examined to remove significant outlier observations for each phenotype after a Bonferroni correction for α = 0.05. The variance component estimates generated by refitting the full model (Figure 1) for each outlier screened phenotype were used to calculate heritability on a line-mean basis (Holland *et al.* 2003; Hung *et al.* 2012), with the delta method (Lynch and Walsh, 1998; Holland *et al.* 2003) used to calculate their standard errors.

To generate the best linear unbiased predictor (BLUP) values, a best-fit model was selected for each outlier-screened phenotype through iteratively fitting the above full mixed linear model in ASReml-R version 3.0, and retention of only random effect terms found to be significant (α = 0.05) in a likelihood ratio test (Littell *et al.* 2006). The final best-fitted model was used to obtain a BLUP for each elemental phenotype for each inbred line (Supplementary Table S1). Given that elements are not always distributed evenly among seed tissues (*e.g.*, pericarp, endosperm, and embryo) and extreme grain phenotypes could have substantially altered elemental composition (Lombi *et al.* 2009, 2011; Pongrac *et al.* 2013; Baxter *et al.* 2014), 247 inbred lines classified according to Romay *et al.* (2013) and Germplasm Resources Information Network (GRIN; https://www.ars-grin.gov/) as sweet corn, popcorn, or with an endosperm mutation were conservatively removed from the dataset. All of the remaining 1,919 inbred lines had BLUP values for 10 or more of the 11 elemental phenotypes.

### Genotype data processing and imputation

We used target and reference SNP genotype sets in B73 RefGen_v4 (B73 v4) coordinates to increase the marker density of the Ames panel with an approach similar to Ramstein *et al.* (2020). In brief, the raw genotypes of genotyping-by-sequencing (GBS) SNPs (ZeaGBSv27_publicSamples_raw_AGPv4-181023.h5, available on CyVerse at http://datacommons.cyverse.org/browse/iplant/home/shared/panzea/genotypes/GBS/v27) scored at 943,455 loci were obtained for the Ames panel from Romay *et al.* (2013), providing a total of 2,172 GBS samples with a call rate ≥20% from 1,839 of the 1,919 inbred lines with phenotypic data for constructing the target set. We initially used a stringent filtered dataset of 35,082 SNPs [call rate ≥50%, % heterozygosity ≤10%, index of panmixia F_IT_ ≥0.8, and linkage disequilibrium (LD) *r*^2^ ≤ 0.2] derived from the Romay *et al.* (2013) dataset to calculate pairwise identity by state (IBS) between multiple samples of the same “accession number” for each of 260 lines in PLINK version 1.9 (Purcell *et al.* 2007). This analysis resulted in the detection and removal of all samples of 23 inbred lines that had a mean IBS value < 0.95 for all within-line sample comparisons, producing a concordance-enhanced dataset of 2,098 GBS samples from 1,816 inbred lines that segregated for biallelic SNPs at 477,155 of the 943,455 SNP loci. To merge two or more GBS samples from the same line, SNP genotype calls with ≥50% occurrence were selected as the consensus genotype, whereas calls with <50% occurrence were set to missing. After consensus-calling, 1,813 lines with a call rate ≥0.2, % heterozygosity ≤10%, and inbreeding coefficient (F) ≥0.8 were retained, which comprised the final set of lines used for downstream genetic analyses. Finally, heterozygous genotype calls were set to missing given their potential to be the result of paralogous alignments.

To construct the reference SNP genotype set, we used the maize HapMap 3.2.1 unimputed datasets (hmp321_agpv4_chrx.vcf.gz, where x is 1 to 10, available on CyVerse at https://datacommons.cyverse.org/browse/iplant/home/shared/panzea/hapmap3/hmp321/unimputed/uplifted_APGv4/) consisting of ∼83 million variants identified from more than 1,200 lines (Bukowski *et al.* 2018) that included variants called from the higher coverage sequencing (average of ∼7x) of the maize 282 (Goodman-Buckler) panel of Flint-Garcia *et al.* (2005). This dataset was processed in the following manner: selection of 14,613,169 SNPs [biallelic, call rate ≥ 50%, minor allele frequency (MAF) ≥1%, local LD flag present, and NI5 flag absent], heterozygous genotype calls set to missing, and imputation of all missing SNP genotype data. With the resultant dataset serving as the reference panel, SNP genotypes at the 14,613,169 loci were imputed based on GBS SNPs (target set) in the final set of 1,813 lines from the Ames panel with BLUP values (Supplementary Table S1). All imputation was conducted in BEAGLE v5.0 (Browning *et al.* 2018) with 10 iterations for initial burn-in, 15 sampling interactions, an effective population size of 50,000 (Ross-Ibarra *et al.* 2009), and the U.S. maize NAM genetic linkage map (McMullen *et al.* 2009) (https://www.maizegdb.org/data_center/map) to provide further information on the recombination landscape. The quality of the imputed genotypes was further enhanced by retaining only biallelic SNPs with MAF ≥1% and predicted dosage *r*^2^ (DR2) ≥ 0.80, resulting in 12,211,420 SNPs. In PLINK version 1.9 (Purcell *et al.* 2007), this SNP dataset was LD pruned with a sliding window of 100 SNPs and step size of 25 SNPs to construct datasets for the 1,813 lines that included only those SNPs with pairwise *r*^2^ < 0.99 (7,719,799 SNPs for marker-trait association tests) or *r*^2^ < 0.10 (361,302 SNPs for population structure and relatedness estimation).

### Genome-wide association study

We conducted marker-trait associations at the genome-wide level as previously described in Owens *et al.* (2019). Briefly, to reduce heteroscedasticity and nonnormality of the residuals, the Box-Cox power transformation procedure (Box and Cox 1964) was invoked for each phenotype with an intercept-only model through the “boxcox” function in MASS package version 7.3-50 in R version 3.5.1 (R Core Team 2018) that chose the optimal convenient lambda (Supplementary Table S2) for the transformation of BLUP values (Supplementary Table S3). With a mixed linear model that used the population parameters previously determined approximation (Zhang *et al.* 2010), each of the 7,719,799 SNP markers was tested for an association with transformed BLUP values of each phenotype from the 1,813 lines in the R package GAPIT version 2018.08.18 (Lipka *et al.* 2012). The fitted mixed linear models included principal components (PCs) and a genomic relationship matrix (kinship) to control for population structure and relatedness. In GAPIT, the 1,813 line × 361,302 SNP genotype matrix was used to calculate the kinship matrix with VanRaden’s method 1 (VanRaden 2008) and PCs with the prcomp function from the R base package. The Bayesian information criterion (Schwarz 1978) was used to determine the optimal number of PCs for model inclusion. The amount of phenotypic variation explained by a SNP was approximated as the difference between the likelihood-ratio-based *R*^2^ statistic (*R*^2^_LR_) (Sun *et al.* 2010) of a mixed linear model with or without a given SNP included. The Benjamini–Hochberg procedure (Benjamini and Hochberg 1995) was used to control the false discovery rate (FDR) at the 5% level for each phenotype.

To better clarify complex association signals, the multi-locus mixed-model (MLMM) approach (Segura *et al.* 2012) that employs forward–backward stepwise regression to sequentially add significant markers as fixed effects (covariates) was used to control for major-effect loci in genome-wide scans for marker-trait associations. The multiple-Bonferroni criterion (mBonf) was used to choose the optimal model. The statistical control of major-effect loci was further evaluated by reconducting GWAS with the MLMM-selected SNPs included as covariates in the mixed linear models fitted in GAPIT.

### Population structure analysis

We classified the inbred lines of the Ames panel to better understand the allele frequency patterns of associated SNPs across subpopulations. The 1,813 line × 361,302 SNP genotype matrix, which had been also used for a principal component analysis (PCA; see Genome-wide association study section of Materials and Methods), served as the input dataset for the estimation of population structure with fastSTRUCTURE (Raj *et al.* 2014). The number of ancestral populations (K) were varied from 1 to 10 with the simple prior when conducting the fastSTRUCTURE analysis. We selected *K* = 3 as the number of subpopulations based on the collective evaluation of the fastSTRUCTURE and PCA results in combination with earlier findings on patterns of population structure in the Ames panel (Romay *et al.* 2013). The 1,813 lines were assigned to one of three subpopulations (SP1, SP2, or SP3) if they had an assignment value of Q ≥ 0.7. If lines had assignment values of Q < 0.7 for all three subpopulations, they were considered to be admixed (Supplementary Table S4). The SP1, SP2, and SP3 subpopulations predominantly consisted of lines classified as nonstiff stalk (NSS), tropical, and stiff stalk (SS), respectively.

### Candidate gene identification

To identify candidate genes, we first constructed a set of distinct loci significantly associated with the elemental phenotypes. A locus was defined as an association signal composed of at least two SNPs significant at 5% FDR within 100 kb from one another, with the most significant SNP designated as the peak marker at a locus. Estimates of pairwise LD (*r*^2^) between a peak SNP and all SNPs within ± 5 Mb were calculated in TASSEL v5.2.49 (Bradbury *et al.* 2007). If two or more peak SNPs occurred within ± 5 Mb of each other, a locus was declared distinct if its peak SNP had an *r*^2^ value < 0.2 with all other designated peak SNPs. The genomic search space to identify candidate genes was limited to within ±100 kb of each peak SNP, given the rapid LD decay in this maize association panel (Romay *et al.* 2013). In addition, the candidate gene search process was partly informed by a curated list of genes involved in the accumulation of elements in plants (Whitt *et al.* 2020). The top three unique best hits of the nine most plausible candidate genes in Arabidopsis (Columbia-0 ecotype) and rice (*O. sativa* L. ssp. Japonica cv. “Nipponbare”) with *E*-values <1 were identified by BLASTP with default parameters at TAIR (https://www.arabidopsis.org) and RAP-DB (https://rapdb.dna.affrc.go.jp) databases, respectively (Supplementary Table S5).

### Integration of genetic mapping results

The genetic mapping results from joint linkage (JL) analysis and GWAS of grain elemental phenotypes in the U.S. nested association mapping (NAM) panel (Ziegler *et al.* 2017) were joined with those generated from our GWAS in the Ames panel (Supplementary Tables S6 and S7). Given that the four field sites (New York, Florida, North Carolina, and Puerto Rico) included in the study of Ziegler *et al.* (2017) had climates and soil types different from those of the Indiana field site, we focused the comparative on NAM genetic mapping results based on BLUP phenotypes generated from a combined analysis of all four locations (All Locs). To accomplish this, first the markers used for JL analysis (SNPs) and GWAS (SNPs and small indels) in the NAM panel were uplifted from B73 v2 to B73 v4. To uplift markers, 50 nt flanking sequences (101 nt total) were clipped from each side of the marker position in the B73 v2 assembly, followed by alignment of the flanking sequences to the B73 v4 assembly through the use of Vmatch v2.3.0 (Kurtz 2010) with the following options: -d -p -complete -h1. Alignments to B73 v4 were filtered to retain the highest scoring and unique alignment for each individual marker. Markers not having a high confidence, unique alignment were discarded from the uplifted results. If markers defining the upper or lower bounds of a QTL support interval could not be uplifted to B73 v4, then the next closest outer SNP marker that could be uplifted was used so as not to compromise the calculated 95% support interval.

### Whole-genome prediction

We evaluated two WGP models, Bayesian ridge regression (BRR) and BayesB (Habier *et al.* 2011; Pérez and de los Campos 2014). The BGLR package version 1.0.8 (Pérez and de los Campos 2014) was used to implement the two WGP models for the transformed BLUP values of each phenotype from the 1,813 lines with a Markov chain Monte Carlo process as follows: 12,000 iterations with a burn-in of 4,000 and a thinning of 5. As a compromise between model run time and performance, the LD-pruned (*r*^2^ < 0.10) dataset of 361,302 genome-wide SNPs was used for both computationally intensive WGP models with an expected minimal loss of information. A stratified fivefold cross-validation scheme that accounted for population structure was conducted 10 times for each of the 11 phenotypes, with predictive ability calculated as the mean Pearson’s correlation of transformed BLUP values with genomic estimated breeding values across folds. Both models used the same fold assignments, and each fold had the same subpopulation (SP) proportion (SP1, SP2, SP3, and admixed) as calculated for the entire panel (Supplementary Table S4).

## Results

### Phenotypic variation

On average, K, P, and Mg were the most abundant (>1,000 μg g^−1^) elements in grain from the Ames panel, followed by Ca at an almost two orders of magnitude lower average concentration (Table 1). For the other elements, the average concentrations of Zn and Fe were closest to Ca, whereas Mn, Cu, B, Mo, and Ni had average concentrations <7 μg g^−1^. The calculated correlations between the 11 elements ranged from essentially nonexistent (*r *<* *0.01) between Mo and Ni to very strong (*r *=* *0.70) between P and Mg (Supplementary Figure S1). Interestingly, we detected a strong correlation (*r *=* *0.55) between Fe and Zn, which suggests that these two elements could have a partially shared genetic architecture. All 11 elements showed significant genotype-by-year interaction, but which accounted for only a small percentage of the total phenotypic variance (Figure 1). The 11 phenotypes had an average heritability of 0.70, with a range of 0.33 for B to 0.87 for Cu (Table 1). Even though these phenotypes were influenced by the environment, our results indicate that the exhibited phenotypic variation was mostly attributable to genetic variation among inbred lines.

**Table 1:** Means, ranges, and standard deviations (Std. Dev.) of untransformed BLUP values (in μg g^−1^) for 11 elemental grain phenotypes evaluated in the Ames panel and estimated heritability on a line-mean basis and their standard errors (Std. Err.) across 2 years

		BLUPs			Heritabilities	
Phenotype	Number of lines	Mean	Range	Std. Dev.	Estimate	Std. Err.
B	1812	2.19	1.59–3.09	0.21	0.33	0.03
Ca	1813	39.72	8.60–121.08	12.7	0.77	0.01
Cu	1812	2.32	0.91–5.75	0.68	0.87	0.01
Fe	1810	23.59	14.62–36.33	3.29	0.75	0.01
K	1813	4435.72	2944.20–6671.02	431.62	0.76	0.01
Mg	1813	1334.16	955.20–1814.08	115.97	0.61	0.02
Mn	1812	6.12	2.38–11.69	1.44	0.78	0.01
Mo	1812	0.49	0.29–0.85	0.07	0.65	0.02
Ni	1809	0.23	−0.04–1.12	0.14	0.77	0.01
P	1813	3298.76	2453.00–4341.12	277.24	0.61	0.02
Zn	1813	30.68	12.59–52.32	4.36	0.79	0.01

### Genome-wide association study

**Figure 2:**
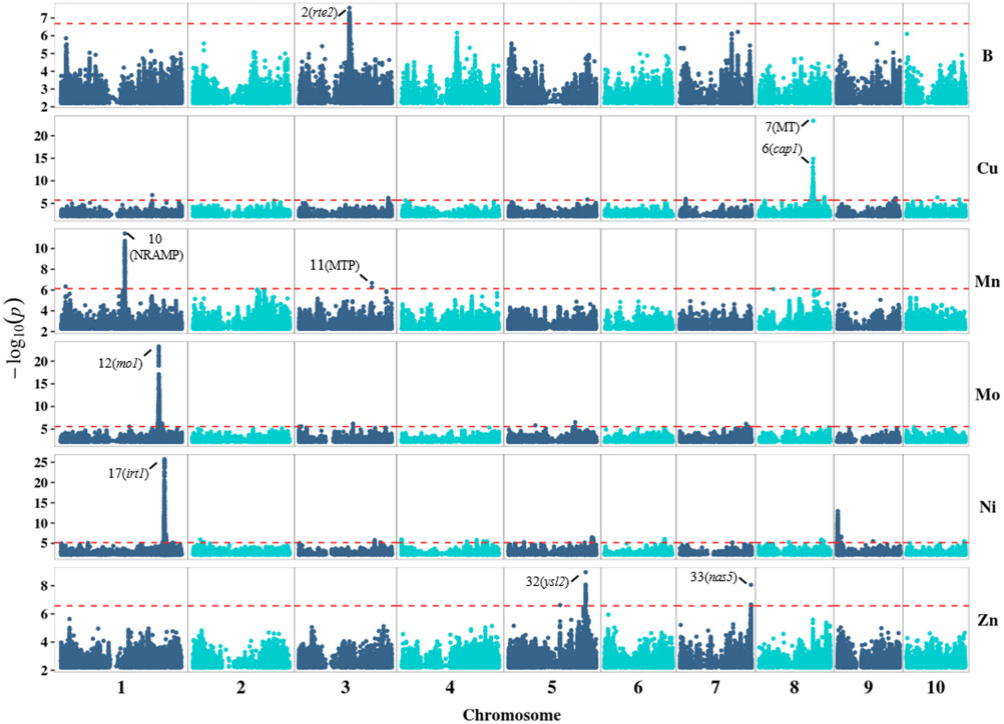
Manhattan plot of results from a genome-wide association study of the six elemental grain phenotypes with significant associations at the 5% FDR level in the Ames panel. Each point represents a SNP with its −log_10_ *P*-value (*y*-axis) from a mixed linear model analysis plotted as a function of physical position (B73 RefGen_v4) across the 10 chromosomes of maize (*x*-axis). The red horizontal dashed line indicates the −log_10_ *P*-value of the least statistically significant SNP at 5% FDR. The most probable candidate genes within ± 100 kb of the most significant SNP (*i.e.*, peak SNP) of each numbered locus are labeled above their corresponding association signals.

A GWAS was conducted for the 11 elemental phenotypes with 1,813 lines of the Ames panel imputed with ∼7.7 million SNPs. Collectively, 1,917 significant marker-trait associations were detected for B, Cu, Mn, Mo, Ni, and Zn, but none were found for Ca, Fe, Mg, K, and P at a genome-wide FDR of 5% (Figure 2 and Supplementary Figure S2). Examination of local LD patterns resolved the 1,917 marker-trait associations into a robust set of 33 loci (Supplementary Table S8). The search space for candidate genes was defined as ±100 kb of the most significant SNP (*i.e.*, peak SNP) at each of the 33 loci, a window size considerate of high marker density, wide variance in rapid rate of LD decay (mean *r*^2^ of 0.2 within ∼1–10 kb) in the panel (Romay *et al.* 2013), and distant cis-regulatory variants (Salvi *et al.* 2007; Studer *et al.* 2011; Wallace *et al.* 2014; Rodgers-Melnick *et al.* 2016; Huang *et al.* 2018; Ricci *et al.* 2019).

**Figure 3:**
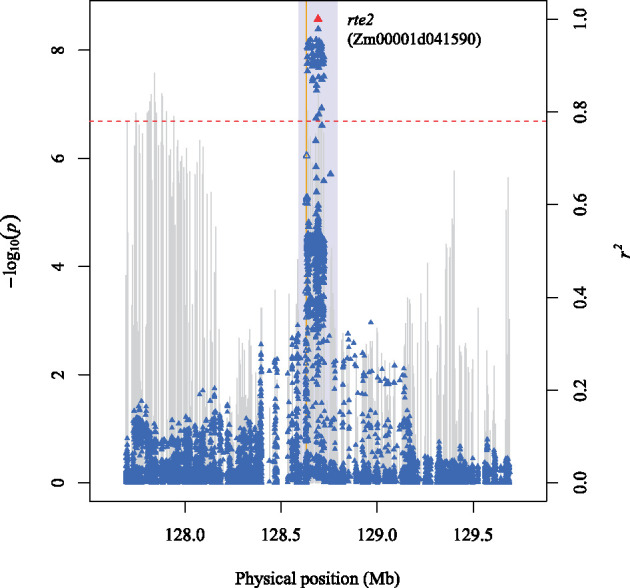
A regional Manhattan plot of locus 2. Scatter plot of association results from a mixed model analysis of B grain concentration and linkage disequilibrium (LD) estimates (*r*^2^) for a genomic region that contains the peak SNP (3-128693026) at locus 2. Each vertical line represents the −log_10_ *P*-value of a SNP. Triangles are the *r*^2^ values of each SNP relative to the peak SNP (indicated in red) at 128,693,026 bp (B73 RefGen_v4) on chromosome 3. The red horizontal dashed line indicates the −log_10_ *P*-value of the least statistically significant SNP at a genome-wide false discovery rate of 5%. The yellow vertical line indicates the genomic position of the *rotten ear2* (*rte2*) gene Zm00001d041590. The open triangles indicate SNPs that are within the candidate gene. The light blue rectangle demarcates the ± 100 kb candidate gene search space surrounding the peak SNP.

The two loci significantly associated with B comprised a mildly complex association signal spanning from 127.4 to 128.7 Mb on chromosome 3 (Figures 2 and 3; Supplementary Table S8). The peak SNP of each locus (locus 1: 3-127841465, *P*-value 2.68E-08; locus 2: 3-128693026, *P*-value 6.47E-08) was separated by a physical distance of ∼851 kb, with virtually no LD (*r*^2^ = 0.03) between them. The peak SNP of the second locus, 3-128693026, was located ∼59 kb from the open reading frame (ORF) of the *rotten ear2* (*rte2*) gene (Zm00001d041590) encoding a B efflux transporter (Chatterjee *et al.* 2017).

The peak SNPs for the strongest two of five association signals for Cu on chromosome 8 (Figure 2) were separated by a physical distance of ∼1.1 Mb and in weak LD (*r*^2^ = 0.15) with each other. Of the two, the more significant peak SNP (8-137939692; *P*-value 4.69E-24) was located within a gene (Zm00001d011063) (Supplementary Figure S3) coding for a protein 43%–60% identical at the amino acid sequence level to three type 2 metallothioneins (MTs) in rice (Supplementary Table S5) (Zhou *et al.* 2006; Kumar *et al.* 2012). Members of the plant MT family are low-molecular weight, cysteine-rich metal-binding proteins and of which some have been shown to bind Cu and other metal ions (Guo *et al.* 2008; Benatti *et al.* 2014).

The least significant of the two peak SNPs (8-136857539; *P*-value 9.10E-15) resided within the *calcium pump1* (*cap1*) gene (Zm00001d011013) (Supplementary Figure S4) encoding a calmodulin-regulated P-type Ca^2+^-ATPase that had been shown to have slightly enhanced mRNA expression in maize roots under anoxic conditions (Subbaiah and Sachs 2000). Although plausible, to our knowledge it had never been reported to transport Cu. The peak SNPs for the other three loci (3–5) on chromosome 8, as well as the two loci (8–9) on chromosomes 3 and 7 were within ±100 kb of candidate genes (Supplementary Table S9) less likely to be involved in Cu chelation or transport.

Of the two loci associated with Mn (Figure 2), the strongest signal was located 162.9 to 163.2 Mb on chromosome 1 (Supplementary Figure S5). The peak SNP (1-162962818, *P*-value 3.61E-12) of this locus resided about 2.2 kb from a gene (Zm00001d030846) encoding a protein with 74% and 72% sequence identity to NRAMP3 and NRAMP4 of Arabidopsis (Supplementary Table S5) that in addition to Fe, export Mn from vacuoles to chloroplasts in leaf mesophyll cells (Lanquar *et al.* 2005, 2010). An additional four SNPs within this gene were significantly associated (*P*-values 7.48E-11 to 3.18E-10) with Mn and in very strong LD (*r*^2^ > 0.90) with the peak SNP.

The weaker effect locus at ∼184.6 Mb on chromosome 3 (Supplementary Figure S6) for Mn was defined by two significant SNPs. Both SNPs were in very strong LD (*r*^2^ = 0.79) with one another. The peak (3-184559931; *P*-value 2.11E-07) and second SNPs (3-184590243; *P*-value 5.46E-07) were approximately 29 and 0.78 kb, respectively, from a gene (Zm00001d042939) that codes for a protein with 80% sequence identity to METAL TOLERANCE PROTEIN 11 (MTP11) of Arabidopsis (Supplementary Table S5) that transports Mn^2+^ (Delhaize *et al.* 2007).

The strongest signal for Mo spanned from 246.5 to 250.3 Mb on chromosome 1 (Supplementary Figure S7), with the peak SNP (1-248672716; *P*-value 5.58E-24) ∼71 kb from the *molybdate transporter1* (*mo1*) gene (Zm00001d033053) that codes for a protein having 69% sequence identity to the mitochondrial-localized MOLYBDATE TRANSPORTER 1 (MOT1) from Arabidopsis (Supplementary Table S5) that specifically transports Mo (Tomatsu *et al.* 2007; Baxter *et al.* 2008). Furthermore, the peak SNP was in very strong LD (*r*^2^ = 0.95) with a highly significant SNP (*P*-value 1.03E-21) located within the gene. The other four loci (13–16) collectively consisted of 10 significant SNPs across three chromosomes but were within ± 100 kb of less probable candidate genes (Supplementary Table S9).

**Figure 4:**
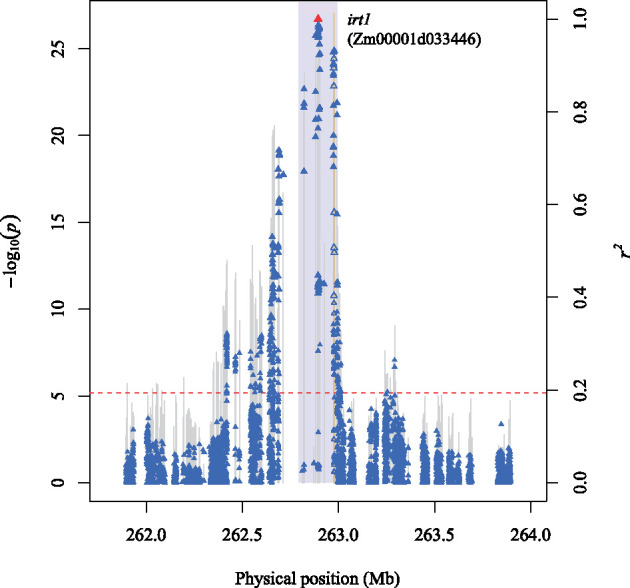
A regional Manhattan plot of locus 17. Scatter plot of association results from a mixed model analysis of Ni grain concentration and linkage disequilibrium (LD) estimates (*r*^2^) for a genomic region that contains the peak SNP (1-262893725) at locus 17. Each vertical line represents the −log_10_ *P*-value of a SNP. Triangles are the *r*^2^ values of each SNP relative to the peak SNP (indicated in red) at 262,893,725 bp (B73 RefGen_v4) on chromosome 1. The red horizontal dashed line indicates the −log_10_ *P*-value of the least statistically significant SNP at a genome-wide false discovery rate of 5%. The yellow vertical line indicates the genomic position of the *iron-regulated transporter1* (*irt1*) gene Zm00001d033446. The open triangles indicate SNPs that are within the candidate gene. The light blue rectangle demarcates the ± 100 kb candidate gene search space surrounding the peak SNP.

Of the 15 loci associated with Ni, the strongest signal mapped from 261.8 to 263.3 Mb on chromosome 1 (Figure 2). The peak SNP (1-262893725, *P*-value 1.98E-26) at this locus (Figure 4) was located ∼82 kb from the *iron-regulated transporter1* (*irt1*) gene (Zm00001d033446) (Mondal *et al.* 2014), which encodes a protein sharing amino acid sequence similarity (55–57% identical) with members of the ZIP transporter family in Arabidopsis that transport a variety of divalent metal ions including Ni^2+^ (Vert *et al.* 2009; Nishida *et al.* 2011; Li *et al.* 2019). In addition, 15 significant SNPs within *irt1* were associated with Ni and, on average, were in strong LD (mean *r*^2^ of 0.52) with the peak SNP. The peak SNPs for the other 14 Ni-associated loci (18–31), however, were within ± 100 kb of candidate genes (Supplementary Table S9) with more speculative involvement in Ni accumulation.

Two significant SNPs comprised the locus associated with Zn at ∼179.9 Mb on chromosome 7 (Supplementary Table S8). Also, these two SNPs were in moderately strong LD (*r*^2^ = 0.69) with each other. Of these two SNPs, the peak SNP (7-179962589; *P*-value 8.67E-09) was nearest (∼1.9 kb) to the *nicotianamine synthase5* (*nas5*) gene (Zm00001d022557) that codes for a class II NAS purportedly involved in synthesizing the metal ion chelator nicotianamine (Zhou *et al.* 2013). Notably, a weaker association signal significant at 15% FDR was identified for Fe with a peak SNP (7-180077496; *P*-value 1.06E-07) at a distance of ∼112 kb from *nas5* (Supplementary Figure S8). The minor allele of each peak SNP, which was associated with a higher mean concentration of either Zn or Fe, occurred at very low frequencies in the tropical (∼5%) and Stiff Stalk (∼1%) subpopulations (Supplementary Table S10).

On chromosome 5, the association signal for Zn ranged from 195.6 to 195.8 Mb (Supplementary Figure S9), with the peak SNP (5-195765640; *P*-value 1.10E-09) only 1.2 kb from the *yellow stripe-like2* (*ysl2*) gene (Zm00001d017427). The protein encoded by *ysl2* has 58% and 63% sequence identity with AtYSL1 and AtYSL3 (Yordem *et al.* 2011) that transport metal-nicotianamine complexes to various Arabidopsis plant tissues (Waters *et al.* 2006). Also, three significant SNPs (*P*-values 7.72E-08 to 1.38E-07) within this gene were in moderately strong LD (mean *r*^2^ of 0.39) with the peak SNP.

### Clarification of association signals to identify the largest-effect loci

The MLMM approach, which helps resolve complex association signals, selected one or more SNPs for Cu, Mn, Mo, Ni, and Zn, but none for the presumably weaker signals of the other six elements (Supplementary Table S11). The top one or two most significant peak SNP markers that had been detected with the mixed linear model in GAPIT were selected by the MLMM for Cu (locus 7; MT), Mn (locus 10; NRAMP), Mo (locus 12; *mo1*), Ni (locus 17; *irt1*), and Zn (locus 32, *ysl2*; locus 33, *nas5*). Furthermore, the MLMM had selected an additional two SNPs, 9-1875785 (locus 29) and 9-745061 (locus 28), for Ni. When a conditional mixed linear model analysis was conducted separately for Cu, Mn, Mo, Ni, and Zn with their respective MLMM-selected SNPs as covariates, there were no remaining significant SNPs at a genome-wide 5% FDR (Supplementary Figure S10). This suggests the MLMM had identified and conditional models had accounted for the major loci contributing to phenotypic variation.

### Comparison of genetic mapping results

We assessed the findings of our study through a comparison involving the JL-QTL analysis and GWAS results of these elemental grain phenotypes in the U.S. maize NAM panel (Supplementary Tables S6 and S7) (Ziegler *et al.* 2017). Of the identified candidate genes, *rte2* and *irt1* were novel associations, whereas the other seven candidate genes were coincident with NAM JL-QTL (Ziegler *et al.* 2017). However, *cap1*, *mo1*, Zm00001d011063 (MT), Zm00001d030846 (NRAMP), *nas5* for Zn, and *ysl2* were distant from their respective NAM GWAS peak variants within JL-QTL support intervals (average: ∼1.68 Mb; range: 0.356–4.67 Mb), whereas *nas5* for Fe and Zm00001d042939 (MTP) were 126.9 and 0.203 kb, respectively, from their closest peak NAM GWAS variant. The joint consideration of GWAS results suggests that the large-effect loci associated with natural variation for the six-grain elements in the NAM panel were all resolved to the level of highly probable causal genes in the Ames panel.

### Whole-genome prediction

We evaluated the predictive ability of WGP for the 11 elemental phenotypes with two models that have different assumptions about the distribution of underlying genetic effects, BRR and BayesB (Habier *et al.* 2011; Pérez and de los Campos 2014). On average, BRR and BayesB had nearly identical prediction abilities of 0.45 and 0.46, respectively, across the 11 phenotypes (Table 2). As expected, given the results of Combs and Bernardo (2013), the predictive abilities of both WGP models were strongly correlated with the heritabilities of all phenotypes (BRR, *r *=* *0.66, *P*-value < 0.05; BayesB, *r *=* *0.65, *P*-value < 0.05). While the predictive abilities from both models were essentially equivalent for most phenotypes, the predictive abilities of Ni, Mo, and Cu increased by 10.42%, 4.00%, and 3.92%, respectively, with BayesB relative to BRR.

**Table 2:** Most plausible candidate genes identified through a genome-wide association study of 11 elemental phenotypes in grain from the Ames panel

Phenotype	Locus number	SNP ID^a^	*P*-value	FDR-adjusted *P*-value	SNP *R*^2b^	Gene ID	Annotated gene function
B	2	3-128693026	6.47E-08	4.59E-02	0.01	Zm00001d041590	B transporter (*rte2*)
Cu	6	8-136857539	9.10E-15	2.34E-08	0.03	Zm00001d011013	Ca transporter (*cap1*)
Cu	7	8-137939692	4.69E-24	3.62E-17	0.04	Zm00001d011063	Metal chelator (MT)
Mn	10	1-162962818	3.61E-12	2.79E-05	0.02	Zm00001d030846	Metal transporter (NRAMP)
Mn	11	3-184559931	2.11E-07	1.71E-02	0.01	Zm00001d042939	Metal transporter (MTP)
Mo	12	1-248672716	5.58E-24	4.31E-17	0.04	Zm00001d033053	Mo transporter (MOT; *mo1*)
Ni	17	1-262893725	1.98E-26	6.75E-20	0.05	Zm00001d033446	Metal transporter (ZIP; *irt1*)
Zn	32	5-195765640	1.10E-09	8.51E-03	0.02	Zm00001d017427	Metal-NA transporter (YSL; *ysl2*)
Zn	33	7-179962589	8.67E-09	1.75E-02	0.01	Zm00001d022557	Metal chelator (NAS; *nas5*)
Fe		7-180077496	1.06E-07	1.53E-01	0.01	Zm00001d022557	Metal chelator (NAS; *nas5*)

a SNP ID nomenclature consists of chromosome number, followed by physical position (bp) in B73 RefGen_v4 coordinates

b SNP *R*^2^ is calculated as follows: *R*^2^ likelihood ratio of model with SNP minus *R*^2^ likelihood ratio of model without SNP (Supplementary Table S8)

## Discussion

Elemental homeostasis is critically important, with prolonged deficiencies or toxicities in essential elements negatively affecting plants (Marschner 2011). To date, the identification of causal genes via GWAS has mostly centered on elemental levels in roots and shoots for model and crop plant species (Huang and Salt 2016; Yang *et al.* 2018), thus the prioritization of candidate genes contributing to elemental accumulation in grain of staple crops remains an important pursuit. To this end, we conducted GWAS on the concentrations of 11 elements in grain from ∼2,000 lines of the maize Ames panel imputed with ∼7.7 million SNP markers. By leveraging the tremendous genetic diversity and rapid intragenic LD decay of this powerful genetic resource, we identified nine candidate genes encoding proteins with functions relevant to the accumulation of elements in maize grain. We also demonstrated moderate prediction abilities for the 11 elements with two different WGP models, which is especially relevant for Fe and Zn biofortification of maize grain (Welch and Graham 2002).

### Novel loci associate with B and Ni

We detected novel associations of *rte2* and *irt1* with levels of B and Ni in maize grain, respectively. The *rte2* gene, coding for a B efflux transporter, is one of six members of a small gene family (*rte1*-*6*) (Chatterjee *et al.* 2017). Even though the duplicated *rte1* and *rte2* genes were reported to have contrasting tissue-specific expression patterns across maize reproductive and root tissues, it was also shown that they work in concert to provide B for maize plants growing in B-depleted soils (Chatterjee *et al.* 2017). It is possible that *rte1* (maize1 subgenome) and *rte2* (maize2 subgenome) functionally diverged following the most recent tetraploidization event of the maize genome (Schnable *et al.* 2011), potentially explaining why not even a very weak association signal was detected with B at *rte1*. Given that *rte2* has high sequence similarity to the class I B transporters of Arabidopsis and rice (Miwa *et al.* 2006; Nakagawa *et al.* 2007; Miwa and Fujiwara 2010; Chatterjee *et al.* 2014, 2017), we hypothesize in our study that *rte2* had an indirect involvement in the accumulation of B in grain by playing a role in xylem loading of B.

The *irt1* gene, which underpinned an association signal for Ni on chromosome 1, is in the maize gene family with sequence similarity to the ZIP family of transporters (Mondal *et al.* 2014) that transport Fe, Zn, and other divalent metal ions in other plants (Eide *et al.* 1996; Grotz *et al.* 1998; Korshunova *et al.* 1999; Li *et al.* 2019). AtIRT1, which is 55% identical in amino acid sequence to ZmIRT1 (Mondal *et al.* 2014), is a plasma membrane protein demonstrated to be critical for Fe^2+^ uptake inside Arabidopsis root epidermal cells (Vert *et al.* 2002), but also showed to have enhanced activity as a transporter of Ni^2+^ in Arabidopsis roots under Ni excess conditions (Nishida *et al.* 2011, 2015). Li *et al.* (2015) reported that overexpression of *Zmirt1* in Arabidopsis produced higher concentrations of Fe and Zn in roots and seeds. Therefore, we speculate that *irt1* contributed to Ni accumulation in maize grain as a metal transporter with a yet to be characterized broader range of specificity that includes Ni^2+^.

### Higher mapping resolution afforded by the Ames panel

The other seven identified candidate genes co-localized with NAM JL-QTL and GWAS signals. With the exception of Zm00001d042939 (MTP), they were more finely mapped in the Ames panel than in the U.S. NAM panel. The proteins with the highest identity (80% and 93%) to Zm00001d042939 in Arabidopsis (AtMTP11) and rice (OsMTP11) (Supplementary Table S5) are Golgi-localized Mn transporters involved in conferral of Mn tolerance by a mechanism hypothesized to involve one or both of vesicular transport to the vacuole or extracellular secretion (Delhaize *et al.* 2007; Peiter *et al.* 2007; Farthing *et al.* 2017; Zhang and Liu 2017; Tsunemitsu *et al.* 2018). Notably, through a GWAS in a sorghum association panel, a syntenic ortholog (Sobic.003G349200) of Zm00001d042939 (Y. Zhang *et al.* 2017) was implicated in the genetic control of Mn grain levels (Shakoor *et al.* 2016). Although different in cellular function and localization compared to MTP11, Zm00001d030846 (NRAMP), a member of a largely uncharacterized maize gene family (Jin *et al.* 2015), encodes a protein closely related to the multispecific metal transporters AtNRAMP3 and AtNRAMP4 in Arabidopsis. In addition to their roles as vacuolar iron effluxers, these two NRAMP proteins were shown by Lanquar *et al.* (2005, 2010) to be functionally redundant vacuolar membrane-localized transporters involved in the export of Mn to the cytosol from the vacuole of mature leaf mesophyll cells in Arabidopsis.

The *mo1* gene, inferred to be orthologous to MOT1 proteins in Arabidopsis and rice (Supplementary Table S5), underlied the Mo association signal on chromosome 1. *AtMOT1* was the first cloned and characterized Mo-specific transporter in plants (Tomatsu *et al.* 2007; Baxter *et al.* 2008) and hypothesized to regulate Mo content (Baxter *et al.* 2008). Complementation studies with Arabidopsis ecotypes also showed that natural allelic variants of *AtMOT1* altered shoot Mo content (Baxter *et al.* 2008). Comparatively, a QTL identified for the genetic control of shoot and grain Mo concentration in a rice mapping population was fine mapped to a molybdate transporter (*OsMOT1; 1*), with the Mo transport activity of this causal gene confirmed via genetic and transgenic complementation (Huang *et al.* 2019). Furthermore, Huang *et al.* (2019) showed that a knockout of *OsMOT1; 1*, a gene shown to have strong root expression, produced lower levels of Mo in grain, resulting likely from lower root-to-shoot translocation of Mo.

The stronger of two association signals for Cu on chromosome 8 was underlain by the candidate Zm00001d011063, which encodes an uncharacterized protein possessing weak amino acid sequence similarity to Arabidopsis MTs (Supplementary Table S5) that are involved in homeostasis and remobilization of Cu (Benatti *et al.* 2014). Although not yet implicated in Cu accumulation, the rice protein with the highest sequence identity to Zm00001d011063, *OSMT2b* (also named as *OsMT-I-2c*) (Supplementary Table S5), had altered transcript abundance in rice shoot and root seedling tissues after Cu treatment (Yuan *et al.* 2008). The second genetically distinct signal coincided with *cap1*, a gene that codes for a calmodulin regulated P-type Ca^2+^-ATPase (Subbaiah and Sachs 2000). The CAP1 protein is 80% identical in sequence to ECA1 in Arabidopsis (Supplementary Table S5), which is an ER-localized P_2A_-type Ca^2+^-ATPase reported to transport Ca^2+^, Mn^2+^, and potentially other divalent cations in root cells (Wu *et al.* 2002). This is a somewhat unexpected but still plausible finding, given that heavy metal P_1B_-type ATPase subfamily members from Arabidopsis and rice have demonstrated Cu transport activity (Hirayama *et al.* 1999; Andrés-Colás *et al.* 2006; Kobayashi *et al.* 2008; Deng *et al.* 2013; Huang *et al.* 2016).

### A key step toward the biofortification of maize grain

Suggestive of a pleiotropic locus for two correlated phenotypes, *nas5* underpinned the coincident association signals for Fe and Zn on chromosome 7. This gene family member encodes a class II NAS putatively responsible for synthesizing nicotianamine–a divalent metal chelator responsible for the internal transport of trace metals including Fe and Zn (Reviewed in Curie *et al.* 2009; Schuler *et al.* 2012). Nicotianamine is also a precursor for producing mugineic acid family phytosiderophores exuded by roots of graminaceous plants to facilitate Fe uptake (reviewed in Curie *et al.* 2009; Swamy *et al.* 2016). In particular, activation tagging of *OsNAS3*, the rice protein with the highest sequence identity to *nas5* (Zhou *et al.* 2013), resulted in higher nicotianamine levels that led to increased Fe and Zn in rice grain (Lee *et al.* 2009). In maize, *nas5* was found to be strongly expressed in stems and induced under excessive Fe and Zn conditions, suggesting its more localized involvement in homeostasis and transport, but this has yet to be extensively characterized (Zhou *et al.* 2013). Nonetheless, the identification of SNPs tagging the low-frequency *nas5* alleles associated with increasing Fe or Zn grain levels is a key step toward facilitating biofortification of tropical maize. Many people with deficiencies for both of these elements subsist predominantly on maize grain in developing nations (Welch 2002; Welch and Graham 2004).

The *ysl2* gene associated with Zn on chromosome 5 encodes a protein with amino acid sequence similarity to the YSL family of transporters that uptake metal-phytosiderophores or metal-nicotianamine complexes (reviewed in Curie *et al.* 2009). Of the three Arabidopsis proteins (AtYSL1-3) with high sequence identity to *ysl2*, AtYSL1 and AtYSL3 (Yordem *et al.* 2011) were both implicated in the remobilization of Zn from senescing leaves as a complex with nicotianamine to developing seeds (Waters *et al.* 2006). Recently, Zang et al. (2020) showed that ZmYSL2 is a metal-nicotianamine transporter involved in the transport of Fe from the endosperm to embryo in the developing maize grain, but importantly they also implicated ZmYSL2 in the transport of Zn. Interestingly, the *ys1* gene that encodes a Fe(III)-PS transporter (Curie *et al.* 2001), the gene family member most closely related to *ysl2* (Yordem *et al.* 2011), was ∼68 kb from the peak SNP for Zn on maize chromosome 5, but has contradictory support as a key contributor for Zn uptake or allocation (Wiren *et al.* 1996; Roberts *et al.* 2004; Schaaf *et al.* 2004; Chan-Rodriguez and Walker 2018). Therefore, *ys1* and *ysl2* merit joint consideration in future fine mapping and mutagenesis studies to more conclusively determine their independent or collective contribution to Zn accumulation in grain.

### Generalizability of genetic mapping results

Importantly, our GWAS findings for all 11 elemental traits may not be generalizable beyond the Ames panel itself or where it was grown. As an example, the number of JL-QTL detected by Ziegler *et al.* (2017) for each of the 11 elemental grain phenotypes ranged from 3 (B) to 17 (Mn) with varied effect sizes (*R*^2^ = 0.8 to 37.6%) in the U.S. NAM panel that affords higher statistical power (Yu *et al.* 2008). The findings of Ziegler *et al.* (2017) include a total of 11, 5, 12, and 11 JL-QTL identified for Ca, K, Mg, and P, respectively, which are the four elements that lacked significant associations in the Ames panel (Supplementary Table S6). Although these detected genetic differences could be attributed to environmental factors that influence elemental accumulation in grain (Ziegler *et al.* 2017), it is also possible that in the Ames panel the genetic architecture for each of these high concentration macroelements is predominated by rare variants of weak to modest effect, thus limiting their detectability even with a high density of SNP markers used in GWAS. Despite the genetic differences between association panels for macronutrients, seven of the nine candidate causal genes identified for micronutrients in the Ames panel co-localized with NAM JL-QTL and GWAS signals, thus these genetic signals are more likely to be reproduced in further independent genetic mapping panels and environments.

### Informing whole-genome prediction with genetic mapping results

We conducted WGP of grain elemental phenotypes in maize, resulting in, on average, moderate predictive abilities from BRR (0.45) and BayesB (0.46) across all phenotypes that are comparable to those obtained for elemental phenotypes in wheat grain (Velu *et al.* 2016; Manickavelu *et al.* 2017; Alomari *et al.* 2018) and for Zn in maize grain (Guo *et al.* 2020; Mageto *et al.* 2020). For Ni, Mo, and Cu, the BayesB model that allows for a few of many genome-wide markers to have large effects (Meuwissen *et al.* 2001; Gianola *et al.* 2009; de Los Campos *et al.* 2013) modestly outperformed (3.92–10.42%; Table 2) the BRR model with homogeneous shrinkage across all markers (Gianola 2013; de Los Campos *et al.* 2013). These three elements had the highest number of associated loci (5–15) and the largest amount of phenotypic variation explained by peak SNPs (3–5%) tagging a candidate causal gene (Figure 2; Table 3), implying that BayesB could better fit the genetic architecture of Ni, Mo, and Cu (de Los Campos *et al.* 2013). Taken together, our GWAS-informed WGP results provide a foundational framework for exploring the additional modeling of identified large-effect loci when conducting genomic selection of elemental grain phenotypes in maize breeding populations (Bernardo 2014).

**Table 3:** Predictive abilities of 11 elemental grain phenotypes of the Ames panel from Bayesian ridge regression (BRR) and BayesB models

Phenotype	BRR		BayesB	
	Predictive ability	Std. Dev.	Predictive ability	Std. Dev.
B	0.33	0.01	0.33	0.01
Ca	0.47	0.01	0.47	0.01
Cu	0.51	0.01	0.53	0.01
Fe	0.46	0.01	0.46	0.01
K	0.34	0.01	0.34	0.01
Mg	0.45	0.01	0.45	0.01
Mn	0.50	0.01	0.50	0.01
Mo	0.50	0.01	0.52	0.01
Ni	0.48	0.01	0.53	0.01
P	0.40	0.01	0.40	0.01
Zn	0.50	0.01	0.50	0.01

## Conclusions

We found 11 elemental grain phenotypes to be moderately heritable in the maize Ames panel, with minor but significant genotype-by-year interaction. The novel associations of *rte2* and *irt1* with B and Ni, respectively, in combination with enhanced pinpointing of seven candidate casual genes for Cu, Fe, Mn, Mo, and/or Zn illustrate the high level of statistical power and mapping resolution conferred by the Ames panel for genetically dissecting complex trait variation in maize. However, not all detected GWAS signals were resolved down to an individual gene with a definitive role in metal transport or chelation, thus potentially revealing novel candidate genes that could be further assessed for function in mutagenesis experiments. In addition, we identified two loci (*nas5* and *ysl2*) that could be leveraged with marker-based breeding approaches to increase Zn levels in maize grain. Notably, the *nas5* gene also associated with the concentration of Fe in grain, thus helping to enable multi-trait selection (Jia and Jannink 2012) for developing biofortified maize varieties to help combat dietary Fe and Zn deficiencies that collectively affect more than 2 billion people worldwide (Viteri 1998; Prasad 2014). Furthermore, the moderate WGP prediction accuracies for Zn and Fe concentrations imply that both grain phenotypes should respond favorably to genomic selection approaches. Overall, our work has provided new insights into the genetic architecture of elemental accumulation in maize grain and strengthened the knowledge base needed to accelerate genomics-assisted breeding efforts for increased grain concentrations of Zn and Fe in maize breeding populations.

## Data availability

The raw genotypes of GBS SNPs (ZeaGBSv27_publicSamples_raw_AGPv4-181023.h5) are available on CyVerse (at http://datacommons.cyverse.org/browse/iplant/home/shared/panzea/genotypes/GBS/v27). The maize HapMap 3.2.1 unimputed datasets (hmp321_agpv4_chrx.vcf.gz, where x is 1 to 10) are available on CyVerse (at https://datacommons.cyverse.org/browse/iplant/home/shared/panzea/hapmap3/hmp321/unimputed/uplifted_APGv4/). The untransformed and transformed BLUP values of the phenotypes are provided in Supplementary Tables S1 and S3, respectively. Supplemental Material available at figshare: https://doi.org/10.25387/g3.13644098.
